# Human placental oxygenation in late gestation: experimental and theoretical approaches

**DOI:** 10.1113/JP275633

**Published:** 2018-02-25

**Authors:** Gareth A. Nye, Emma Ingram, Edward D. Johnstone, Oliver E. Jensen, Henning Schneider, Rohan M. Lewis, Igor L. Chernyavsky, Paul Brownbill

**Affiliations:** ^1^ Maternal and Fetal Health Research Centre, Division of Developmental Biology and Medicine, School of Medical Sciences, Faculty of Biology, Medicine and Health, University of Manchester Manchester Academic Health Science Centre Manchester M13 9WL UK; ^2^ St Mary's Hospital, Central Manchester University Hospitals NHS Foundation Trust Manchester Academic Health Science Centre Manchester M13 9WL UK; ^3^ School of Mathematics University of Manchester Manchester M13 9PL UK; ^4^ Department of Obstetrics and Gynecology, Inselspital University of Bern CH‐3010 Bern Switzerland; ^5^ Faculty of Medicine University of Southampton Southampton SO16 6YD UK

**Keywords:** placenta, oxygen, perfusion, MRI, intervillious space, FGR, modelling

## Abstract

The placenta is crucial for life. It is an ephemeral but complex organ acting as the barrier interface between maternal and fetal circulations, providing exchange of gases, nutrients, hormones, waste products and immunoglobulins. Many gaps exist in our understanding of the detailed placental structure and function, particularly in relation to oxygen handling and transfer in healthy and pathological states *in utero*. Measurements to understand oxygen transfer *in vivo* in the human are limited, with no general agreement on the most appropriate methods. An invasive method for measuring partial pressure of oxygen in the intervillous space through needle electrode insertion at the time of Caesarean sections has been reported. This allows for direct measurements *in vivo* whilst maintaining near normal placental conditions; however, there are practical and ethical implications in using this method for determination of placental oxygenation. Furthermore, oxygen levels are likely to be highly heterogeneous within the placenta. Emerging non‐invasive techniques, such as MRI, and *ex vivo* research are capable of enhancing and improving current imaging methodology for placental villous structure and increase the precision of oxygen measurement within placental compartments. These techniques, in combination with mathematical modelling, have stimulated novel cross‐disciplinary approaches that could advance our understanding of placental oxygenation and its metabolism in normal and pathological pregnancies, improving clinical treatment options and ultimately outcomes for the patient.

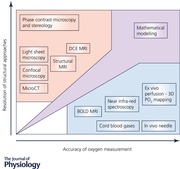

## Introduction

The placenta is vital for fetal growth and development, adapting its physiology, architecture and signalling throughout gestation to meet changing demands. Despite this, there remain many unanswered questions in our detailed understanding of placental structure, function and transfer efficacy in both normal and diseased states *in utero*.

It is an ephemeral but complex organ acting as the interface between mother and fetus, providing a hub for exchange. One of the key functions of the placenta is to mediate transfer of oxygen to the fetus. However, there is poor consensus on the oxygenation of placental compartments, most notably oxygen gradients within the intervillous space (IVS) of the maternal circulation, and how the partial pressure of this gas ($P_{O_{2}}$) differs between normal pregnancies and those complicated by placental diseases. A full understanding of spatio‐temporal oxygenation and associated placental villous architecture in healthy and diseased states, aiding mathematical model development on transplacental oxygen transfer, will ultimately be useful to obstetricians trying to understand and treat placental disease. In this review, we will present current views on human placenta structure and function with respect to oxygen transfer. This will include discussions on the strengths and weaknesses of the current methods used to measure placental oxygenation both *in vivo* and *ex vivo*. It will also summarise reported oxygen levels within the placenta–fetal unit, with an emphasis on dysregulated materno‐fetal oxygen transfer in pregnancy pathologies.

## Placental structure and function

The human placenta is a discoid haemomonochorial dually perfused organ, which in a healthy term pregnancy has a mean mass of 650 g and a surface area for exchange of 13 m^2^ (Mayhew *et al*. 2007). It contains 25% (80 mL) of the total fetal blood volume (Luckhardt *et al*. 1996). Fetal blood flow from the two umbilical arteries is forced through two elaborately branched networks across the chorionic plate before delving into the placental mass where they branch again entering approximately 50 placental villous trees. Several villous trees might occupy a single lobule, which is the semi‐compartmentalised structure defined by septa as seen from the basal plate aspect. Villous trees are elaborately branched, commencing with stem villi (Leiser *et al*. 1985). Stem villi divide extensively to form intermediate villi, with the mature type branching off to form the terminal villi (Kaufmann *et al*. 1985). In later pregnancy, having arrived at the villous capillaries via an arteriolar microcirculatory system, the fetoplacental capillary blood enriched with oxygen and nutrients is forced into the venular systems of the mature intermediate and stem villi, and then into veins on the chorionic plate, subsequently travelling to the fetus via a single umbilical vein. Architectural events unfold in the developing placenta throughout gestation to arrive at the position of mature villous trees capable of servicing the ever‐increasing demand of the fetus for oxygen. See e.g. Huppertz (2008) and Wang and Zhao (2010) for the detailed anatomy and physiology of the human placenta and its developmental aspects.

**Figure 1:**
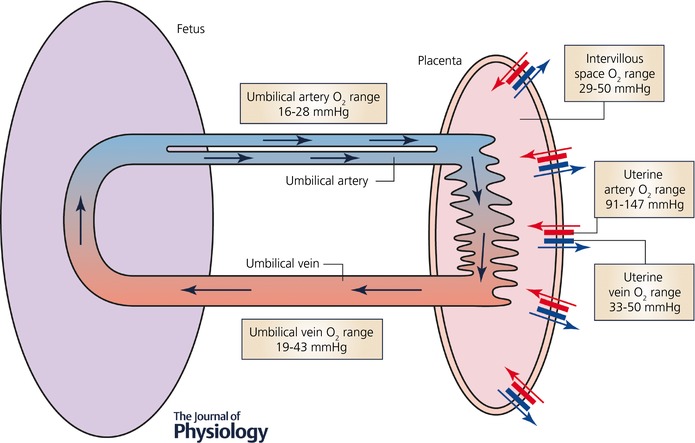
Simplified schematic diagram of the maternal and fetal placental circulations, showing the major compartments and published attributed *in vivo* oxygen values (see Table 1)

During the third trimester, terminal villi formation increases exponentially (Risau & Rubanyi, 2000). From 24 to 26 weeks of gestation, branching angiogenesis ensues, leading to capillary outgrowths and the maturation of intermediate villi. Capillary loops are hypothesised to dilate and remodel laterally under transmural hydrostatic pressure between the fetal and maternal placental circulations (Burton *et al*. 1996). Furthermore, fetal and maternal blood are brought into close proximity at specialised adaptive capillary structures known as vasculosyncytial membranes. These represent a thinning of the combined fetoplacental endothelium and syncytialised trophoblast, with extensive lateral displacement of single‐celled trophoblasts and membrane‐associated organelles (Castellucci *et al*. 1990). This structural adaptation confines the endo‐epithelial placental barrier to a diffusion distance of 2–3 μm, an important facet of Fick's law of diffusion, appertaining more particularly to the efficiency of transfer of hydrophilic molecules (Sibley *et al*. 1998), but also in part to fast diffusing gases like oxygen. The pathway of blood between the placenta and fetus with compartmentalised reported oxygen ranges in late pregnancy has been summarised for the purpose of this review in Fig. 1.

Although the placenta is involved in essential functions to maintain fetal health, little is known about how human placenta transfer *in vivo* relates to fetal oxygen acquisition in the human. The placenta is dynamic, with the capability of adapting to possible net reductions in maternal blood flow, ensuring there is an adequate supply of oxygen to the fetus (Wilkening & Meschia, 1983). *In vivo* work using animal models, most notably the sheep, shows a normal tolerance to reduced maternal‐side placental blood flow, before placental metabolic demands out‐compete fetal demands for oxygen provision from the maternal circulation (Gu *et al*. 1985). The work of Gu, who collaborated with Professor Julian Parer whilst he was at the University of California, showed that a reduction in uterine artery blood flow by up to 30% had no effect on fetal oxygenation levels (Gu *et al*. 1985). Whilst the anatomy of the sheep placenta is substantially different to the human placenta, broader concepts such as hypoxic fetoplacental vasoconstriction and the possible role of oxygen‐sensitive voltage‐gated potassium channels in this process, as found in humans, might provide a cross‐species mechanism by which feto‐maternal blood flow matching could arise (Byrne *et al*. 1997; Hampl *et al*. 2002; Kiernan *et al*. 2010).

Modern *in vivo* imaging, *ex vivo* placental spatial oxygen mapping technology and mathematical modelling are now available to investigate these early observations and aim to unravel the intricacies of how fetal oxygen acquisition is regulated by placental structure and function in health and disease. This multidisciplinary approach has shown the impact of sinusoidal capillaries on placental function (Pearce *et al*. 2016; Plitman Mayo *et al*. 2016b) where mathematical modelling indicates the existence of an optimal capillary dilatation size that maximises oxygen uptake. A fuller understanding of the remaining aspects of the placental oxygen transfer in health and disease now seems possible.

## Oxygen metabolism and levels within the human placenta

In the placenta, gases diffuse due to partial pressure gradients which are maintained by maternal and fetal blood flow. As diffusion of gases across the placenta is rapid, placental gas transfer is flow limited (Meschia *et al*. 1967). Oxygen transfer depends on a partial pressure gradient being present between the maternal blood in the intervillous space (IVS) and the fetal blood in the fetoplacental capillaries and is enhanced by the Bohr–Haldane effect: as maternal blood takes up fetal carbon dioxide and becomes acidotic, oxygen release to the fetus is favoured. Simultaneously fetal blood takes up oxygen while decreasing its storage capacity for carbon dioxide and releasing it into the maternal circulation (Pinnock *et al*. 2002). Additionally, there is a higher affinity of the fetal haemoglobin for oxygen, fetal haemoglobin containing two α and two γ subunits compared to maternal haemoglobin which has two α and two β subunits. When considering oxygen levels within the placenta, the differing compartments need to be viewed separately. The normal range for oxygenation of adult blood is between 75 and 100 mmHg. Although there may be a slight reduction in this during pregnancy, it is not expected to differ greatly. IVS soluble oxygenation values are expected to be reduced by the metabolic demand of placental tissue and the transfer to the fetal circulation. It is known that 40% of the total oxygen consumption occurs in the syncytiotrophoblast layer (Carter, 2000). However, where there is failure of spiral arteries to transform to a wider aperture, this potentially leads to altered haemodynamics, with a reduced net IVS flow, more localised blood flow patterns, and disparate skewed oxygen gradients around placental villi (Burton *et al*. 2009). This in turn ultimately leads to placental dysfunction and disease.

## Poor placental oxygenation – a trigger for placental dysfunction

The placenta is developed to maximise the transfer of gases and nutrients to aid the growth of the fetus. When this fails, a wide range of maternal and fetal complications can occur, one of the most common complications being fetal growth restriction (FGR) where the fetus fails to reach its genetic growth potential. Over half of neonatal deaths worldwide are associated with low birth weight (UNICEF, 2004). Surviving FGR neonates face developmental problems and an increased risk of cardiovascular diseases in later life.

There are many causes of FGR involving both maternal and placental factors (Sharma *et al*. 2016). One key factor is a reduction in oxygen transfer to the baby. Placentas measured from FGR babies are on average 24% smaller in weight than normal pregnancies (Heinonen *et al*. 2001). In the absence of genetic abnormalities and underlying maternal conditions, this suggests a reduced functional capacity of the placenta. One hypothesised aetiology is a reduced placental surface area for gas exchange, coupled with dysregulated placental blood flow, leading to suboptimal oxygen transfer from maternal to fetal circulations (Yu, 1992). An alternative aetiology is that dysregulated placental morphology might also reduce the oxygen transfer across the placenta. Non‐placental aetiologies relate to maternal lifestyle factors which include smoking, living at high altitude and heart or lung disease, all of which depress the $P_{O_{2}}$ in the maternal circulation, diminishing placental oxygen transfer (Sharma *et al*. 2016). In such low oxygen environments, as found in some cases of FGR, it should be borne in mind that placental metabolism might shift to a high glucose and low oxygen consumption mode, which could have a bearing on relative oxygen transfer rates to the fetus. This indirect evidence comes from an analogous study of high altitude pregnancies, referring to a reduced maternal oxygen supply to the placenta (Zamudio *et al*. 2010).

Conversely, Kingdom *et al*. proposed processes whereby changing oxygen levels can alter the structure of capillarisation within the terminal villous tree (Kingdom *et al*. 2000). Potentially this can compound an already compromised disturbance in $P_{O_{2}}$, further reducing oxygenation of the fetal circulation. The hypothesis remains to be tested that compromised villous tree architecture coupled with existing reduced oxygen levels in the maternal circulation exceeds a critical threshold leading to FGR.

A comprehensive study including mathematical modelling of complex placental architecture, coupled with *ex vivo* physiological perfusion experimentation and *in vivo* magnetic resonance imaging (MRI), will provide further answers. This may then permit an interrogation of transplacental transfer efficacy of oxygen, providing translational tools for obstetricians in their diagnosis and management of FGR associated with oxygen transfer deficiency.

## Current understanding of placental oxygen levels

As discussed previously, the *in vivo* measurement of placental oxygen has proved difficult and has only been recorded in a handful of studies. Schaaps *et al*. published the most recent cross‐sectional study (Schaaps *et al*. 2005) using IVS blood sampling which was achieved through insertion of a 21 gauge needle through the chorionic plate, with blood being collected once the baby was delivered, but before the placenta was expelled (Schaaps *et al*. 2005). This had previously been done in 1960 by Quilligan *et al*. and again in 1996 by Fujikura & Yoshida, who both recorded lower average values. The Schaaps paper also published a $P_{O_{2}}$ ratio between the uterine vein and the IVS of 1.5 (Schaaps *et al*. 2005). Using this ratio, values of 50 mmHg in the uterine vein (Sibley *et al*. 2002) would lead to an approximate IVS value of 33 mmHg. With near infrared spectroscopy, Kakogawa *et al*. (2010) predicted an IVS value of 30 mmHg. Although providing the best‐available estimate of IVS $P_{O_{2}}$, caution must be applied to the uterine vein–IVS ratio extrapolator. The ratio first appears counterintuitive, since uterine vein blood occurs downstream of the IVS. However, arteriovenous placental shunting and preferential IVS flow pathways evading $P_{O_{2}}$ measurement may lead to higher than expected uterine vein oxygen values. Ideally, further ubiquitous IVS real‐time data must be sought before relying solely on this reported ratio.

The few studies recording IVS oxygenation in term placentas show a value of approximately 36 mmHg. This is much lower than the $P_{O_{2}}$ of peripheral maternal arterial blood, which does not drop below 100 mmHg throughout gestation (Templeton & Kelman, 1976), potentially indicating transfer loss and a highly metabolic cellular layer of the IVS.

Compartmentalised *in vivo* values of soluble human placental oxygenation are given in Fig. 1 and corresponding published values are summarised in Table 1. A small reduction in the oxygen levels between the IVS and the umbilical vein is evident with an average IVS oxygen recording of 30 mmHg and a further reduction in values between the umbilical vein and arteries (22 mmHg). However, there is much greater variation in the recorded values in both measures potentially due to different experimental methods. In particular, there are differences in practice regarding clamping of the umbilical cord after delivery. As shown in Table 1, other studies measuring both venous and arterial values from the same cord recorded similar reductions in the arterial values (Nicolaides *et al*. 1989; Link *et al*. 2007). The venous oxygen level recorded by Schaaps *et al*. is somewhat lower than other recorded values and lower than the average value by 10 mmHg, which is possibly due to the measurement of unclamped cords influencing the oxygen value (Schaaps *et al*. 2005). It is expected that cord clamping will yield results closer to peripartum $P_{O_{2}}$ levels, due to cord samples being compartmentalised away from the highly metabolic placental tissue.

**Table 1:** *In vivo* IVS and *ex vivo* umbilical artery and vein $P_{O_{2}}$ values in term normal human placentas

	$P_{O_{2}}$ values^a^ (no. of samples)	Type of measurement	Time of measurement	Reference
Pre‐partum IVS	34 (*n* = 4)	18 gauge needle	Before placental shedding	(Quilligan *et al*. 1960)
	30 (*n* = 12)	21 gauge needle	Before placental shedding	(Fujikura & Yoshida, 1996)
Post‐partum IVS	49 (*n* = 12)	18 gauge needle	N/A	(Haruta *et al*. 1986)
	33 (*n* = 6)	Uterine vein analysis	Post placental shedding	(Sibley *et al*. 2002)
	29 (*n* = 9)	21 gauge needle		(Schaaps *et al*. 2005)
	30 (*n* = 15)	Uterine vein analysis		(Kakogawa *et al*. 2010)
Range	**29–49**			
Weighted mean ± SD	**34 ± 9**			
Umbilical artery	28 (*n* = 53)	Cordocentesis	Before Caesarean section	(Nicolaides *et al*. 1989)
	18 (*n* = 681)	N/A	Post placental shedding	(Dudenhausen *et al*. 1997)
	21 (*n* = 60)	21 gauge needle		(Daniel *et al*. 1998)
	30 (*n* = 18)	Blood gas analyser		(Ochiai *et al*. 1999)
	16 (*n* = 1281)	N/A		(Arikan *et al*. 2000)
	19 (*n* = 46)	N/A		(Link *et al*. 2007)
	26 (*n* = 60)	N/A		(Fardiazar *et al*. 2013)
	23 (*n* = 46)	N/A		(Di Tommaso *et al*. 2014)
Range	**16‐30**			
Weighted mean ± SD	**18 ± 4**			
Umbilical vein	35 (*n* = 14)		Before Caesarean section	(Pardi *et al*. 1987)
	43 (*n* = 143)	Cordocentesis		(Nicolaides *et al*. 1989)
	31 (*n* = 60)	21 gauge needle	Post placental shedding	(Daniel *et al*. 1998)
	18 (*n* = 18)	Blood gas analyser		(Ochiai *et al*. 1999)
	19 (*n* = 9)	21 gauge needle		(Schaaps *et al*. 2005)
	25 (*n* = 46)	N/A		(Link *et al*. 2007)
	29 (*n* = 300)	N/A		(Bernardez‐Zapata & Moreno‐Rey, 2014)
	27 (*n* = 46)	N/A		(Di Tommaso *et al*. 2014)
Range	**19–43**			
Weighted mean ± SD	**32 ± 8**			
Uterine artery	97 (*n* = 50)	Electrode	Post placental shedding	(Blechner *et al*. 1968)
	147 (*n* = 18)	Blood gas analyser		(Ochiai *et al*. 1999)
	91 (*n* = 168)			(Postigo *et al*. 2009)
Range	**91–147**			
Weighted mean ± SD	**97 ± 22**			
Uterine vein	33	Electrode	Post placental shedding	(Stave, 1970)
	50 (*n* = 6)	Blood gas analyser		(Sibley *et al*. 2002)
	46 (*n* = 10)	21 gauge needle	Before placental shedding	(Fujikura & Yoshida, 1996)
Range	**33–50**			

a $P_{O_{2}}$ values are in mmHg. N/A, not available. Cross‐study values are presented as range and mean ± SD, weighted with the sample size.

## Measuring and modelling oxygen transfer function in the human placenta

### Assessing function through *in vivo* electrodes

A reported method for analysing placental oxygen status of the IVS is through the insertion of a needle into the placental tissue during routine Caesarean sections (Quilligan *et al*. 1960; Fujikura & Yoshida, 1996) (Table 1). Although this allows for direct measurement *in vivo* whilst still under normal conditions, there is a sampling efficiency problem due to the limited number of IVS $P_{O_{2}}$ measurements that can realistically be taken during surgery in such a large tissue. The heterogeneity of $P_{O_{2}}$ levels within the IVS and the potential for contamination of IVS samples from disruption of the fetal capillaries are also major problems with this early method. However, there have been recent moves towards *in vivo* techniques to measure oxygen more ubiquitously in the human placenta.

### Assessing function through magnetic resonance imaging

**Figure 2:**
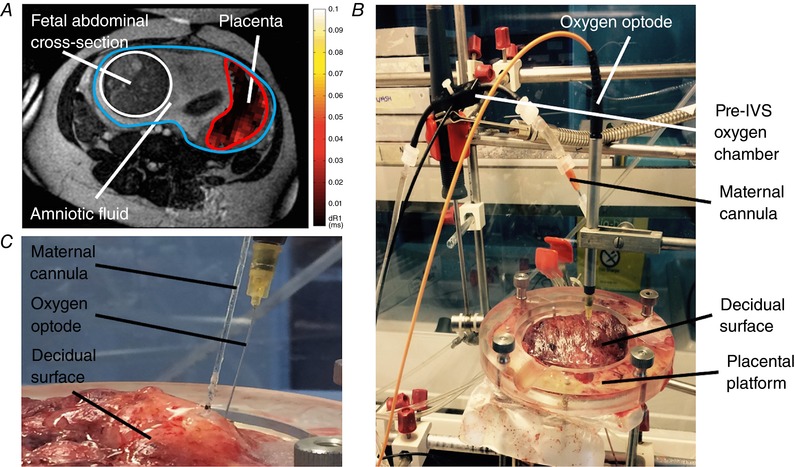
Measuring oxygen distribution in the human placenta *A*, demonstrating a normal placenta imaged using oxygen‐enhanced (OE) MRI techniques. This shows an axial T2‐weighted structural MR image through maternal abdomen at the level of the uterine cavity (circled in blue) demonstrating: fetal abdominal cross‐section (circled in white) and placental region of interest (circled in red) with a superimposed delta R1 map. The colour gradient voxels in the dR1 map demonstrate the differing change in $P_{O_{2}}$ across the placenta following maternal hyperoxia. *B*, soluble oxygen measurement of the perfusate in the maternal‐side arterial inflow line and the IVS in the *ex vivo* dually perfused placental lobule, measured via a pre‐IVS oxygen chamber (flow‐through cell) and via an optode needle inserted into the placental tissue, respectively. *C*, a close‐up image showing the insertion points of both the maternal cannula (i.d. = 2 mm, super‐glued into position to form a seal with the decidua) and the IVS oxygen optode needle inserted through the decidual surface of the placenta.

MRI techniques have demonstrated the ability to measure non‐compartmentalised changes in oxygen levels within defined placental spatial parameters. One such technique, blood‐oxygen‐level‐dependent (BOLD) MRI (Fig. 2 *A*), can effectively measure changes in placental oxygen saturation following a maternal oxygen challenge. Deoxyhaemoglobin acts as an endogenous contrast agent, due to the differing magnetic properties of both oxyhaemoglobin and deoxyhaemoglobin. Changes in oxygen saturation, and therefore deoxyhaemoglobin levels, alter the local magnetic field susceptibility, thus affecting transverse relaxation times and BOLD signal. The first human placental BOLD MRI study (Sorensen *et al*. 2013) described results from eight women with uncomplicated singleton pregnancies at 28–36 weeks’ gestation. An increased BOLD signal was detected in areas proximal to the chorionic plate of the placenta. However, the potential application of BOLD in placental pathology is uncertain with conflicting data concerning FGR pregnancies in early comparative studies (Ingram *et al*. 2017). The interpretations of BOLD signal changes is complex due to its relation to haemoglobin concentration and potential oxygen‐related changes in local perfusion. The signal is also potentially affected by undetected uterine activity and there is a tendency for BOLD signal intensity to be correlated more closely with fetal haemoglobin oxygen saturation than with maternal haemoglobin oxygen saturation. This may be due to the relative hypoxic condition of the normal fetus, which results in a significant BOLD signal change, with changes in oxygen concentration, operating along the exponential phase of the sigmoidal fetal haemoglobin oxygen association curve.

In addition to BOLD MRI, effective changes in tissue $P_{O_{2}}$ have been determined using a complementary technique, oxygen‐enhanced (OE) MRI. In OE MRI, changes in longitudinal relaxation rates (R1) occur due to an increase in the paramagnetic dissolved oxygen content in the tissue with maternal hyperoxia. An increase in R1 following maternal hyperoxia, reflecting an increase in $P_{O_{2}}$, was first demonstrated in the placenta in 2013 (Huen *et al*. 2013). Increases in R1 following hyperoxia diminish with gestational age, which is thought to be a consequence of rapid materno‐fetal O_2_ transfer and utilisation. Additionally, in pregnancies affected by FGR, R1 changes are significantly lower presumably demonstrating a relative placental hypoxia as more of the dissolved oxygen is bound to deoxyhaemoglobin (Ingram *et al*. 2017). The benefit of these techniques are their non‐invasive nature; however, they are limited in availability and expensive (Sorensen *et al*. 2013). Essentially these techniques provide measures of relative change in tissue oxygen status. However, these techniques cannot provide absolute $P_{O_{2}}$ or saturation values without further phantom validation.

Understanding placental blood flow rates is also important in deciphering placental oxygen transfer efficacy. Within *in vivo* imaging capability, several options might be available to the researcher in appreciating flow: dynamic contrast enhanced imaging (DCE) (Marcos *et al*. 1997), arterial spin labelling (ASL) (Gowland *et al*. 1998) and phase contrast imaging (Jansz *et al*. 2010). DCE MRI provides spatial images of villous capillary (fetal) and IVS (maternal) flow. Substances such as Omniscan (a gadolinium chelate) are unstable and therefore potentially toxic when used *in vivo* and have not been characterised for vascular leakage and signal stability. They therefore are only suited for *ex vivo* perfusions or acute animal experiments. These validations are essential in proving that acquired flow signals are truly compartmentalised. The future research agenda in placenta MRI is optimization of acquisition techniques and combining MRI approaches, such as OE with ASL, to fully characterise the placenta.

In ASL, blood is intrinsically labelled, avoiding the concerns of exogenous contrast agents, and therefore this technique has been used *in vivo* to determine placental flow; however, to date, ASL has been performed on a placental region‐of‐interest which incorporates both maternoplacental and fetoplacental compartments (Shao *et al*. 2017). ASL quantifies flow per gram of tissue mass but the technique is hampered by poor signal‐to‐noise ratio and the few studies that have been performed demonstrate considerable variation in derived normal values.

Whilst functional MRI (fMRI) may be of benefit through the measurement of placental perfusion and oxygen status, its use in the placenta is still limited by a lack of data and there are no accepted MRI‐based definitions of normal/abnormal placental tissue flow rates (Avni *et al*. 2015). Again, this could be due to differences in cost and availability but also through a lack of consensus on protocols, and poor image quality due to the challenges of correcting for maternal and fetal motion. However, these MRI techniques could be exploited *ex vivo*, through phantom perfusion investigations, utilising the human dual placental perfusion model to quantify flow and validate *in vivo* perfusion measures, improving our understanding of the imaging response as a proxy to tissue oxygenation.

### Assessing function through *ex vivo* placental perfusion

There is a limited capability to manipulate *in vivo* physiological variables during human pregnancy. In this stance, the utilisation of *ex vivo* physiological research techniques is now coming to the fore. *Ex vivo* dual perfusion of the human placenta is now a widely used system for investigating a range of pharmacological and physiological functions including drug transfer whilst maintaining placental structure and an approximate *in vivo* state (Fig. 2 *B*). Perfusion has advantages over cell culture, tissue slices and explant studies due to the maintenance of villous architecture and relative IVS volume density (Brownbill *et al*. 2018). Vascularised fetoplacental and IVS perfusate flows are key features of the model in which the placental tissue maintains a higher metabolic level than in other human placental models (Hauguel *et al*. 1983). This technique involves isolating a whole placental cotyledon from a freshly delivered placenta. The fetal side is cannulated on both arterial and venous sides and either near‐anoxic blood or physiological buffer is pumped through the villous microcirculation (Schneider & Huch, 1985). The maternal side is also supplied with blood or physiological buffer at normoxic or superoxic $P_{O_{2}}$ levels. [53, 54] Flow rates of perfusate are similar to, but less than, *in vivo* conditions (fetal side, 6 mL min^−1^) to reduce the overall resistance encountered during the experiment. Placental blood flow on the fetal side is calculated to be approximately 0.35 mL min g^−1^ *in vivo* at term, based on the placenta receiving 40% of fetal left ventricular output, being 480 mL min^−1^ at term. This compares to an *ex vivo* fetal‐side flow of 0.17 mL min^−1^ based on a perfused tissue mass of 35 g being perfused at 6 mL min^−1^ (Desforges *et al*. 2017). Once perfusion is established, a number of experiments can be undertaken that are not possible *in vivo*. Examples include increasing or decreasing the flow rate of either fetal or maternal perfusate, introducing drugs such as vasodilators/constrictors, or multiple sampling to monitor perfusate gases. There are disadvantages however: there is a high preparation failure rate; it is reasonably expensive and time‐consuming to run an experiment; and only one lobule from each placenta is usually suitably intact for perfusion, preventing parallel control investigations.

To simplify our understanding of placental oxygen transfer, a new adapted version of this model is being trialled by our laboratory. This involves scaling down the established maternal‐side multi‐cannula dual perfusion model, so that the IVS irrigation volume is limited, employing just one maternal cannula delivering normoxic perfusate and measuring oxygen gradients within the IVS (Fig. 2 *B*). Unlike *in vivo* oxygen sampling, extensive IVS oxygen sampling under steady‐state experimental conditions is possible by means of an oxygen‐sensitive needle optode inserted through the decidual plate at set *X–Y–Z* planes controlled with a micromanipulator (Fig. 2 *B*). With further experimentation the placental metabolic component of IVS oxygen consumption can be elucidated. IVS oxygen gradient data can be acquired and interrogated for metabolism and transfer, and it may be possible to discover how variable perfusate flow rates and fetoplacental vasoactive endocrine agents affect fetal‐side oxygen acquisition, gaining an understanding of how the associated underlying villous architecture enhances or constrains oxygen transfer across the placental barrier.

**Figure 3:**
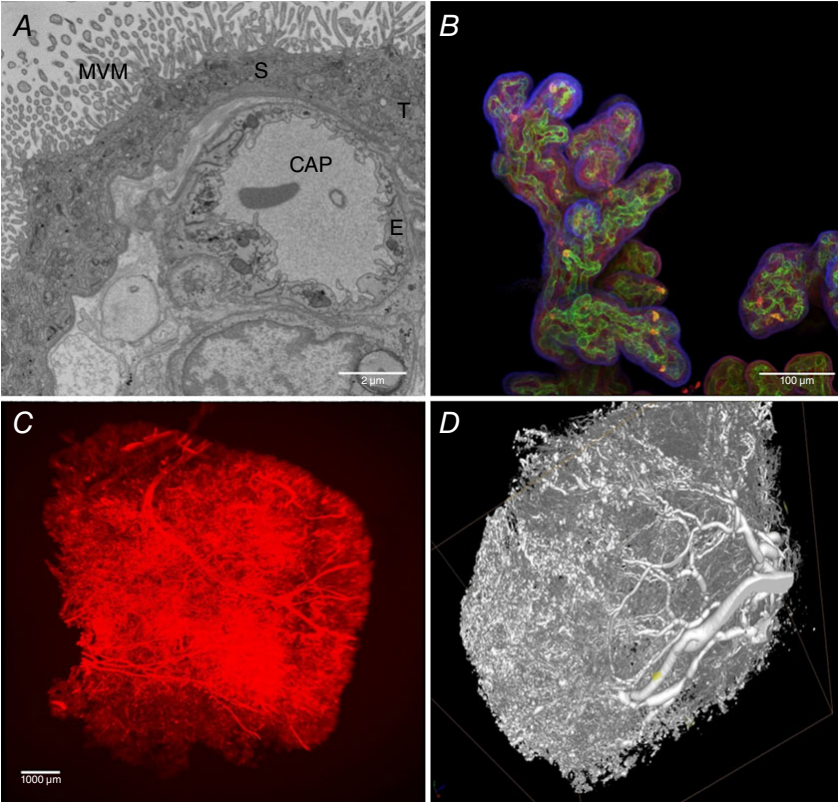
Evaluating imaging techniques for use in assessing placental structure *A*, a transmission electron micrograph of terminal villi showing microvillous membrane (MVM), an underlying capillary (CAP), a syncytiotrophoblast (S), a trophoblast (T) and endothelium (E). *B*, projection of an imaged stack (wholemount confocal microscopy), stained with lectin FITC‐AAL for the endothelium (green), rhodamine‐PSA for the stroma (red) and biotin‐DSL for the trophoblast (violet); the DSL was detected with streptavidin 680 and imaging was on a Leica Sp5 confocal microscope, presented as an image stack. *C*, villous microcirculation of a term normal placenta perfused with a Ulex Europaeus Agglutinin (UEA) lectin linked to biotin and detected with streptavidin 800. *D*, microCT image of a vascular corrosion cast of a term placenta, infused through the umbilical artery with Batson's resin, which was then set, followed by tissue corrosion steps for several days in 20% (w/v) potassium hydroxide.

Post‐perfused human placental tissue can be successfully imaged using wholemount confocal and light sheet microscopy. These 3‐D approaches confer advantages over traditional 2‐D techniques such as transmission‐electron and phase‐contrast microscopy (Fig. 3 *A*), and allow the surface of the villi and the fetal vascular system to be differentially labelled (Fig. 3 *B* and *C*). However, these higher‐resolution approaches are only able to image smaller regions of tissue. Micro‐computed tomography (MicroCT) allows the visualisation of larger regions of placental villi placenta but at different scales (Fig. 3 *D*). Imaging of the fetoplacental vascular system can be enhanced by perfusing contrast agents into the fetal circulation to image the arterial and venous circulation (Junaid *et al*. 2017). From this, information on vessel branching patterns, interbranching length and capillary loop dilatations are useful in predicting the placenta's ability to optimally transfer oxygen, a portion of the placenta often inaccessible by other means (Junaid *et al*. 2017). However, while microCT can image large regions of tissue, when doing this its ability to image the microcirculation is limited. MicroCT imaging of fetoplacental capillaries is possible but in smaller pieces of tissue.

### Integrating structure and function relationships through modelling

**Figure 4:**
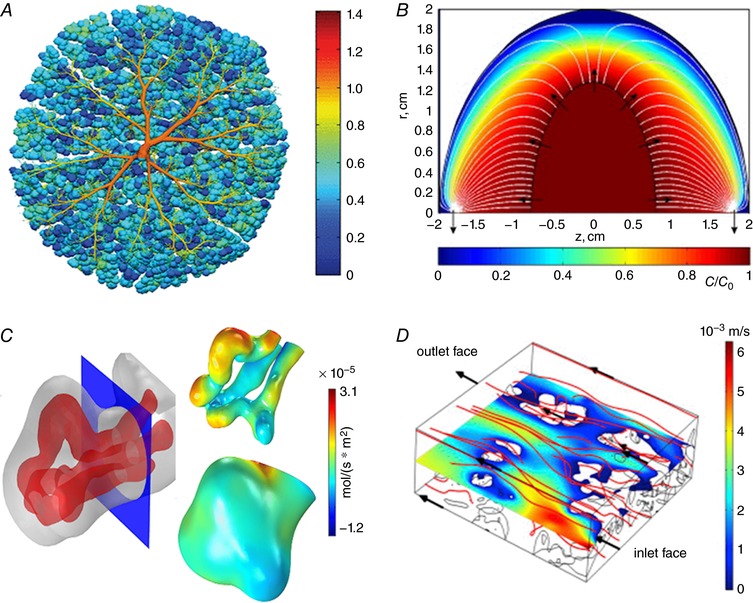
Mathematical modelling of human placental perfusion and oxygenation at different scales *A*, variability of perfusion in a fetoplacental vascular network (colour scale shows pressure for chorionic vessels and relative capillary flow for terminal capillaries, plotted as spheres) (Clark *et al*. 2015). *B*, distribution of a passive solute in the intervillous space of a single placental lobule (Chernyavsky *et al*. 2010). *C*, oxygen flux distribution over the capillary and syncytiotrophoblast surfaces of a single terminal villus (Pearce *et al*. 2016). *D*, microscopic flow in the intervillous space (Perazzolo *et al*. 2017). Images are reproduced with permission, subject to the respective copyrights.

The increased availability of 3‐D imaging approaches, including confocal microscopy and microCT, have made possible the recent increase in efforts to build multiscale computational models that go hand‐in‐hand with refined experimental models (Pearce *et al*. 2016; Plitman Mayo *et al*. 2016a; Perazzolo *et al*. 2017; Roth *et al*. 2017) (Fig. 4). In tissues with complex structures such as the human placenta, computational modelling has allowed the full structure to be visualised and analysed, facilitating assessment of structure–function relationships (Clark *et al*. 2015; Plitman Mayo *et al*. 2016a) (Fig. 4 *A*). Mathematical models have also been created to explain properties of the placenta that it would not be possible to understand using *in vivo* measurements alone (Chernyavsky *et al*. 2011; Serov *et al*. 2015; Pearce *et al*. 2016) (Fig. 4 *B*–*D*). However, there is the open challenge of effective extraction of structural information from imaging data as well as of identifying key parameter values necessary for mathematical modelling. Once validated, theoretical models could provide a bridge between *in vivo* and *ex vivo* or *in vitro* approaches to characterise placental structure and oxygenation in normal and pathological pregnancies (Lecarpentier *et al*. 2016).

## Conclusion

The physiology of placental oxygen transfer is crucial for optimal fetal development and survival. Gross placental structure is well characterised and newer techniques, such as MRI, microCT and advanced microscopy scanning techniques, are affording greater detail. However, the function of the placenta remains poorly understood. Key measurements of oxygen and carbon dioxide levels in a normal human placenta remain elusive due to logistical and ethical complications with experimenting on *in vivo* human placentas, which are compounded by slow advances of *ex vivo*, *in vitro* and *in silico* work.

This lack of fundamental understanding has led to slow progress in terms of treating pathological states such as in cases of fetal growth restriction. It is our theory that impaired placental oxygen transfer and metabolism (Schneider, 2015) may well be a key factor in many cases of FGR; however, whether this is due to abnormal structure or abnormal function is currently unknown. It is only with robust measures and consensus in experimental design that we can develop an integrated understanding of structure–function relationships within the placenta. This in turn will provide a basis for developing therapeutic interventions for the treatment of the placenta in fetal diseases *in vivo*.

## Additional information

### Competing interests

None declared.

### Author contributions

All authors approved the final version of the manuscript and agree to be accountable for all aspects of the work. All persons designated as authors qualify for authorship, and all those who qualify for authorship are listed.

### Funding

Funding was provided by the Medical Research Council, MR/N011538/1.
